# The definition of response and inadequate response to topical corticosteroid treatment in atopic dermatitis and related skin inflammatory diseases: A GA^2^LEN ADCARE statement paper^☆^

**DOI:** 10.1016/j.waojou.2026.101380

**Published:** 2026-04-15

**Authors:** Torsten Zuberbier, Mohamed Abuzakouk, Irena Angelova Fischer, Luisa Karla Arruda, Matthias Augustin, Lisa Beck, Jonathan Bernstein, Carsten Bindslev-Jensen, Marta Bertolín-Colilla, Christine Bangert, Oscar Calderón-Llosa, Giorgio Walter Canonica, Mary Anne Roldan-Castor, Roxanne J. Casis-Hao, Ivan Cherrez-Ojeda, Karen Jui Lin Choo, Leena Chularojanamontri, Michael J. Cork, Timothy J. Craig, Paulo Ricardo Criado, Adnan Custovic, Tubanur Çetinarslan, Furkan Çalıcıoğlu, Marjolein de Bruin-Weller, Nikolaos Douladiris, Luis Felipe Ensina, Yasemin Erdem Öztürk, Ragip Ertaş, Silvia Mariel Ferrucci, Nelson Rosário Filho, Carsten Flohr, Joachim Fluhr, Daria Fomina, Stamatios Gregoriou, Maia Gotua, Ana Maria Giménez-Arnau, Wolfram Hoetzenecker, Carolyn Jack, Roberta Fachini Jardin Criado, Norito Katoh, Alexandros Katoulis, Şefika İlknur Kökçü Karadağ, Desiree Larenas-Linnemann, Haur Yueh Lee, Herminio Lima, Eli Mansour, Charlotte G Mortz, Mara Morelo Rocha Felix, Victor Morris, Ana Moschione Castro, Dayanne Mota Veloso Bruscky, Olga Mukhina, Dinh Van Nguyen, Audrey Nosbaum, Deniz Ozceker, Esen Özkaya, Rabia Oztas Kara, Claudio A.S. Parisi, Jonny Peter, Luis Puig, German Dario Ramon, Catalina Rincón-Pérez, Umit Murat Sahiner, Peter Schmid-Grendelmeier, Esther Serra-Baldrich, Bahar Sevimli Dikicier, Catherine H. Smith, Petra Staubach, Katarina Stevanovic, Ozlem Su Kucuk, Natasa Teovska Mitrevska, Diamant Thaci, Maria Jose Torres, Tiago Torres, Maria Eduarda Trócoli Zanetti, Papapit Tuchinda, Zahava Vadasz, Efstratios Vakirlis, Gleison Vieira Duarte, Hannele Virtanen, Thomas Werfel, Andreas Wollenberg, Margitta Worm, Yik Weng Yew, Manuel P. Pereira

**Affiliations:** a Institute of Allergology, Charité – Universitätsmedizin Berlin, Corporate Member of Freie Universität Berlin and Humboldt-Universität zu Berlin, Berlin, Germany; b Fraunhofer Institute for Translational Medicine and Pharmacology ITMP, Immunology and Allergology, Berlin, Germany; c Cleveland Clinic Abu Dhabi, Abu Dhabi, United Arab Emirates; d Department of Dermatology, Kepler University Hospital, Linz, Austria; e Ribeirão Preto Medical School, University of São Paulo, Ribeirão Preto, Brazil; f University Medical Center Hamburg-Eppendorf, Hamburg, Germany; g Department of Dermatology, University of Rochester, Rochester, USA; h University of Cincinnati, Dept of Medicine, Division of Allergy and Immunology, Cincinnati, OH, USA; i Odense Research Center for Anaphylaxis, Odense University Hospital, Odense, Denmark; j Department of Dermatology and Allergy Center, Odense University Hospital, Odense, Denmark; k Dermatology Department, Hospital del Mar. Hospital del Mar Research Institute, Universitat Autònoma, Universitat Pompeu Fabra, Barcelona, Spain; l Department of Dermatology, Medical University of Vienna, Vienna, Austria; m SANNA Clínica el Golf, San Isidro, Lima, Peru; n Department of Biomedical Science, Humanitas University, Pieve Emanuele, Milano, Italy; o Personalized Medicine, Asthma and Allergy, IRCCS Humanitas Research Hospital, Rozzano, Italy; p Division of Allergy and Immunology Department of Pediatrics College of Medicine - Philippine General Hospital c Manila, Philippines; q University of the Philippines Manila-Philippine General Hospital Division of Allergy & Immunology, Manila, Philippines; r Espiritu Santo University, Samborondon, Ecuador; s Respiralab Research Group, Guayaquil, Ecuador; t Department of Dermatology, Singapore General Hospital, Singapore; u Department of Dermatology, Faculty of Medicine Siriraj Hospital, Bangkok, Thailand; v Sheffield Children's NIHR Commercial Research Delivery Centre, Sheffield, United Kingdom; w Sheffield Dermatology Research, Division of Clinical Medicine, University of Sheffield Medical School, United Kingdom; x Penn State University, Hershey, Pa, Vinmec International Hospital, Times City, Hanoi, Vietnam; y Centro Universitário Faculdade de Medicina do ABC, Santo André, Brazil; z Alergoskin Alergia e Dermatologia SS Ltda, Santo André, Brazil; aa National Heart and Lung Institute, Imperial College London, United Kingdom; ab Manisa Celal Bayar University, Department of Dermatology and Venereology, Manisa, Turkey; ac Health Sciences University, Kayseri Faculty of Medicine, Kayseri City Hospital, Department of Dermatology, Urticaria & Angioedema & Atopic Dermatitis Center of Reference and Excellence Clinic, Turkey; ad University Medical Center Utrecht, the Netherlands; ae Clinical & Molecular Allergology Center, Chalkidiki, Greece; af CPAlpha Clinical Research Center, Barueri, Brazil; ag İstanbul University, İstanbul Faculty of Medicine, Department of Dermatology and Venereology, 34093 Çapa-İstanbul, Turkey; ah Dermatology Unit. IRCCS Ca' Granda Ospedale Maggiore Policlinico Foundation of Milan, Italy; ai Federal University of Parana, Brazil; aj Global Allergy and Asthma European Network (GA2LEN) Urticaria and Angioedema Center of Reference and Excellence (UCARE/ACARE), Moscow City Research and Clinical Center of Allergy and Immunology, City Clinical Hospital 52, Moscow Healthcare Department, Moscow, Russia; ak Department of Clinical Immunology and Allergy, The First Sechenov State Medical University, Moscow, Russia; al Department of Pulmonology, Astana Medical University, Astana, Kazakhstan; am National and Kapodistrian University of Athens, 1st Department of Dermatology, Andreas Sygros Hospital, Athens, Greece; an Center of Allergy and Immunology, UCARE, ADCARE, ACARE and ANACARE Centers, Tbilisi, Georgia; ao David Tvildiani Medical University, Tbilisi, Georgia; ap Dermatology Department, Hospital del Mar. Hospital del Mar Research Institute Universitat Autonoma, Universitat Pompeu Fabra, Barcelona, Spain; aq Affiliation: Department of Dermatology, Kepler University Hospital, Medical Faculty, Linz, Austria; ar Innovaderm Research, Montreal, Quebec, Canada; as North Campus, Kyoto Prefectural University of Medicine, Japan; at 2nd Department of Dermatology and Venereology, National and Kapodistrian University of Athens, Medical School, Attikon General University Hospital, Athens, Greece; au Prof.Dr.Cemil Taşcıoğlu City Hospital, Istanbul, Turkey; av Center of Excellence in Asthma andnAllergy, Médica Sur Clinical Foundation and Hospital, México City, Mexico; aw Senior Consultant, Department of Dermatology, Singapore General Hospital, Singapore; ax Allergy Centre, Singapore General Hospital, Singapore; ay DUKE-NUS Medical School, Singapore; az McMaster University Division of Clinic Immunology and Allergy and Division of Dermatology, Hamilton, ON, Canada; ba Allergy and Immunology, Department of Internal Medicine, School of Medical Sciences, University of Campinas/UNICAMP, Campinas, Brazil; bb Department of Dermatology and Allergy Center, Odense University Hospital, University of Southern Denmark, Odense, Denmark; bc Alergolife Center, Universidade Federal do Estado do Rio de Janeiro, Rio de Janeiro, Brazil; bd St Luke's General Hospital Carlow/Kilkenny, Kilkenny, Ireland; be Clinica Croce, São Paulo, SP, Brazil; bf Unidade de Alergia e Imunologia Instituto da Criança HCFMUSP, São Paulo, SP, Brazil; bg Universidade Federal de Pernambuco, Recife, Brazil; bh Moscow Clinical Hospital 52 Research Center of Allergy and Immunology Moscow, Russia; bi Vinmec-VinUni Institute of Immunology, VinUniversity, Hanoi, Vietnam; bj Center of Allergy and Clinical Immunology, Vinmec Times City Hospital, Vinmec Healthcare System, Hanoi, Vietnam; bk Hospices Civils de Lyon, Service d'Allergologie et Immunologie Clinique, 69495 Pierre Benite cedex, France; bl Université Claude Bernard, Lyon, France; bm Epidermal Immunity and Allergy, Centre International de Recherche en Infectiologie (CIRI), Lyon France; bn Department of Dermatology, Sakarya University Faculty of Medicine, Sakarya, Turkey; bo Hospital Italiano de Buenos Aires, Ciudad de Buenos Aires, Argentina; bp Division of Allergy and Clinical Immunology, Groote Schuur Hospital, Department of Medicine, University of Cape Town, South Africa; bq Allergy and Immunology Unit, University of Cape Town Lung Institute, South Africa; br Department of Dermatology, Hospital de la Santa Creu i Sant Pau, Barcelona, Spain; bs Universitat Autònoma de Barcelona, Barcelona, Spain; bt Institut de Recerca Sant Pau (IR SANT PAU), Barcelona, Spain; bu Hospital Italiano Regional del Sur, Buenos Aires, Argentina; bv Instituto de Alergia e Inmunología del Sur, Buenos Aires, Argentina; bw Unidad de Especialidades Medicas, Universidad del Ejercito y Fuerza Aérea Mexicana, SEDENA, Mexico; bx Pediatric Allergy Department Hacettepe University Childrens Hospital, 06230, Ankara, Turkey; by Allergiestation, Dermatologische Klinik, Universitätsspital, Zürich, Switzerland; bz Dermatologische Abteilung, Hochgebirgsklinik Davos Wolfgang, Medizincampus Davos, Switzerland; ca Christine Kühne Center for Allergy Research and Education CK-CARE, Davos, Switzerland; cb Sakarya University School of Medicine, Department of Dermatology, Sakarya Training and Research Hospital, Sakarya, Turkey; cc Kings College London, United Kingdom; cd Department of Dermatology University Medical Center Mainz Germany, Germany; ce Bezmialem Vakif University School of Medicine Dermatology Department, Adnan Menderes Bulvarı Vatan Street PK 34093 Fatih, İstanbul, Turkey; cf Remedika General Hospital, dermatology department, Skopje, Macedonia; cg International Balkan University, Skopje, Macedonia; ch Institute and Comprehensive Center for Inflammation Medicine, University of Luebeck, Luebeck Germany; ci Allergy Unit, IBIMA-Hospital Regional Universitario de Málaga-ARADyAL, UMA, Málaga, Spain; cj Department of Dermatology, Centro Académico Clínico, ICBAS/Santo António, University of Porto, Portugal; ck BnaiZion Medical Center, Haifa, Israel; cl First Department of Dermatology-Venereology, School of Medicine, Aristotle University, Thessaloniki, Greece; cm IBIS - Instituto Bahiano de Imunoterapia, Brazil; cn Skin and Allergy Hospital, Helsinki University Hospital, Helsinki, Finland; co Department of Dermatology and Allergy, Hannover Medical School, Hannover, Germany; cp Department of Dermatology and Allergy, University Hospital Augsburg, Augsburg, Germany; cq Comprehensive Center for Inflammation Medicine, University of Luebeck, Luebeck, Germany; cr Department of Dermatology and Allergy, Ludwig-Maximilian University, Munich, Germany; cs Division of Allergy and Immunology, Department of Dermatology, Venereology and Allergy, Charité - Universitätsmedizin Berlin, Germany; ct National Skin Centre, Singapore

**Keywords:** Dermatitis, Atopic, Adrenal cortex hormones, Skin diseases, Inflammatory, Delphi technique, Consensus, Topical administration

## Abstract

**Background:**

Topical corticosteroids (TCS) remain the first-line treatment for atopic dermatitis (AD) and related inflammatory skin diseases, yet no standardized definition of response exists. This gap contributes to heterogeneity in clinical practice and complicates trial design. We therefore aimed to develop consensus-based definitions of response and inadequate response to TCS therapy through a structured international eDelphi process.

**Methods:**

A PubMed search (1974–July 2025) identified 403 relevant publications. Candidate statements were drafted from the evidence and refined by the ADCARE Steering Committee, categorized into 3 domains (status quo, unmet need, proposals), and evaluated in a three-round eDelphi survey among certified ADCARE members. Eighty-four dermatologists and allergists from 32 countries participated (Round 1 response rate 98%; Round 2, 80%; Round 3, 76%). Statements were rated on a 5-point Likert scale; consensus was defined as ≥75% agreement (scores 4 or 5).

**Results:**

In total, 66 of 83 statements reached consensus. In the status quo domain, agreement centred on baseline severity, body surface area, and anatomical site as guiding factors for TCS choice, with potency and licensed duration considered central to safe prescribing. In the unmet-need domain, experts highlighted the absence of standardized definitions, variability in monitoring and escalation strategies, and gaps in long-term evidence and integration of patient-reported outcomes. In the proposal domain, consensus supported relative improvement thresholds (≥50% in EASI, SCORAD, itch NRS, IGA, PGA, POEM) and 14 days as a meaningful evaluation point. Absolute cut-offs, very short (7 days) or long (3 months) timeframes, and rigid escalation rules did not achieve consensus. These parameters were synthesized into concise and extended definitions of TCS response and inadequate response.

**Conclusions:**

This GA^2^LEN ADCARE initiative represents the first international consensus on defining TCS response and inadequate response, offering a framework to harmonize clinical practice, enhance trial comparability, and support guideline development.

## Introduction

Topical corticosteroids (TCSs) remain a fundamental component in the therapeutic management of inflammatory skin diseases, particularly atopic dermatitis (AD). They are among the most frequently prescribed medications for dermatologic conditions worldwide and are recommended as first-line therapy in many national and international AD guidelines.^1^, ^2^, ^3^, ^4^, ^5^, ^6^

TCSs exert their therapeutic effects in AD primarily through binding to cytoplasmic glucocorticoid receptors, which translocate to the nucleus and regulate gene transcription.^7^ This suppresses pro-inflammatory cytokine expression, reduces Th2 and dendritic-cell activity, limits eosinophil recruitment, and promotes restoration of epidermal barrier integrity.^8^^,^^9^ Nevertheless, not all patients respond adequately. Impaired penetration, receptor variability, and broader immune dysregulation (not targeted by TCSs) seen in moderate-to-severe AD can result in partial or absent responses.^10^ Patients with mild AD often achieve rapid control of symptoms with short-term use of low-to medium-potency corticosteroids, whereas in patients with moderate-to-severe disease prolonged or repeated courses of potent TCSs, often in combination with adjunctive therapies, are needed.^11^ However, while these molecular and clinical mechanisms explain the efficacy of TCSs, the definition of a meaningful clinical response remains heterogeneous and insufficiently standardized.

In clinical practice, TCSs are prescribed across a spectrum of potencies and formulations, with dosing guided by therapeutic index, licensed duration, and safety considerations. The fingertip unit, defined as the amount of ointment expressed from the distal phalanx of the index finger to the first crease, covering approximately 2% of an adult's body surface area, serves as a practical dosing measure. TCSs are typically applied once or twice daily, with super-high-potency preparations recommended for no longer than 3 weeks, and high- or medium-potency agents for up to 12 weeks. No strict time limits are defined for low-potency corticosteroids.

Despite their widespread use, there is no standardized definition of “treatment response” to TCS. This lack of clarity complicates both clinical practice and the comparability of clinical trials. To explore whether existing trial data could inform a unified definition of TCS response, we collated endpoints from clinical studies in which TCS were used (Table 1).

**Table 1:** Endpoints used in clinical trials to evaluate the response to TCSs in combination with adjunct therapy for AD treatment.

Trial (Population)	Adjunct therapy	TCS regimen	Primary response endpoint(s)	Key secondary endpoints
LIBERTY AD CHRONOS^12^ (adults, moderate–severe)	Dupilumab	Low–medium potency, daily	IGA 0/1 with ≥2-point improvement; EASI-75 at week 16	EASI-75 and IGA 0/1 at week 52; pruritus NRS (≥3–4 pt reduction); QoL scores
NCT03345914^13^ (children, severe)	Dupilumab	Medium potency	IGA 0/1 with ≥2-point improvement; EASI-75	Safety, pruritus, QoL
ADhere^14^ (adolescents & adults, moderate–severe)	Lebrikizumab	Low–medium potency	IGA 0/1 with ≥2-point improvement; EASI-75 at week 16	Itch, sleep, QoL, AEs
AD up^15^^,^^16^ (adolescents & adults, moderate–severe)	Upadacitinib	Unspecified	EASI-75; vIGA-AD 0/1 with ≥2-point improvement at week 16	Pruritus NRS ≥4; sustained responses to week 52
ECZTRA 1–3^17^ (adults, moderate–severe)	Tralokinumab	Mid-strength, daily	IGA 0/1 and/or EASI-75 at week 16	QoL, symptom relief, safety
ARCADIA 1–2^18^ (adolescents & adults, moderate–severe)	Nemolizumab	Low–medium potency	EASI-75; IGA 0/1	Pruritus, QoL
BREEZE-AD PEDS^19^ (children, moderate–severe)	Baricitinib	Low–medium potency	EASI-75; IGA 0/1	Itch, safety
TREBLE^20^ (adults, moderate–severe)	Lebrikizumab	TCS twice daily	EASI-50 at week 12	Symptom relief, safety

Abbreviations: IGA = Investigator's Global Assessment; EASI = Eczema Area and Severity Index; NRS = Numerical Rating Scale; QoL = Quality of Life; vIGA-AD = validated Ivestigator's Global Assessment for Atopic Dermatitis; TCS = Topical Corticosteroids

To address this unmet need, GA²LEN’s ADCARE subnetwork took on the initiative to define standardized, consensus-based criteria for assessing response and inadequate response to TCS therapy. GA²LEN (Global Allergy and Asthma Excellence Network), founded in 2004, is the world’s largest excellence network for asthma and allergy. The ADCARE subnetwork, comprising 67 internationally recognized expert centers, is dedicated to advancing excellence in AD care through clinical management, education, and research. The aim of this initiative is to move beyond subjective assessments toward reproducible, clinically meaningful parameters that can be implemented in both clinical trials and everyday care. To our knowledge, this is the first international initiative to establish standardized definitions of TCS response and inadequate response in AD and related inflammatory skin diseases.

## Methods

### Literature search

A qualitative literature search was conducted in PubMed using the search string [(topical corticosteroid) AND (response) AND (atopic dermatitis)] to gain an overview of how topical corticosteroid response in the treatment of AD is currently defined in a clinical setting. As the objective was to provide a qualitative overview rather than a systematic review, the search strategy was intentionally limited and may not capture all relevant publications. This search yielded 403 publications dating from 1974 to 03.07.2025. Titles and abstracts were screened by 2 reviewers independently for inclusion of clinically applicable definitions or assessment criteria for TCS response in AD treatment.

### Expert panel

All members of the ADCARE network were invited to participate (n = 118) in an online eDelphi survey. Participants were dermatologists and allergists from ADCARE-certified centers, as defined by the GA²LEN ADCARE audit criteria. A total of 84 ADCARE members agreed to participate in the project.

### eDelphi process

Findings from the literature search overview were used by the ADCARE steering committee to identify current TCS response definitions used in clinical trials for AD. Based on these findings and internal discussion, the steering committee drafted the initial set of Delphi statements reflecting the status quo, existing gaps, and potential proposals for standardized response definitions. These statements were circulated to participants in a three-round electronic Delphi (eDelphi) iterative process conducted via Google Forms, ensuring anonymity of responses. Each statement was rated on a 5-point Likert scale (1 = strongly disagree, 5 = strongly agree). The statements were organized into 3 categories: status quo, unmet need, and proposals.

In Round 1, 58 statements were evaluated. Participants were encouraged to provide open comments, which were used to refine, merge, or expand statements, mainly to clarify wording. The agreement thresholds were reached for all statements, however, comments suggested further clarification. Based on this feedback, additional candidate items were introduced, resulting in 83 statements for Round 2. There were no major concerns in round 2. In Round 3, all individual consensus statements were pooled into 2 comprehensive formulations, a concise (*short*) and a detailed (*extended*) version, describing TCS treatment response. In addition, 2 new statements were introduced to assess whether the established parameters for the treatment response to TCSs in AD could, in principle, be extrapolated to other inflammatory dermatological diseases treated with TCSs (eg, hand eczema).

### Consensus definition and data analysis

Consensus was defined as ≥75% of respondents selecting “agree” (4 on the Likert scale) or “strongly agree” (5 on the Likert scale)”. Descriptive analyses were performed in Microsoft Excel. The Likert scale median score and the interquartile range (IQR) were calculated for each item to assess central tendency and response stability. The geographic distribution of participants was visualized using Datawrapper.

## Results

**Fig. 1:**
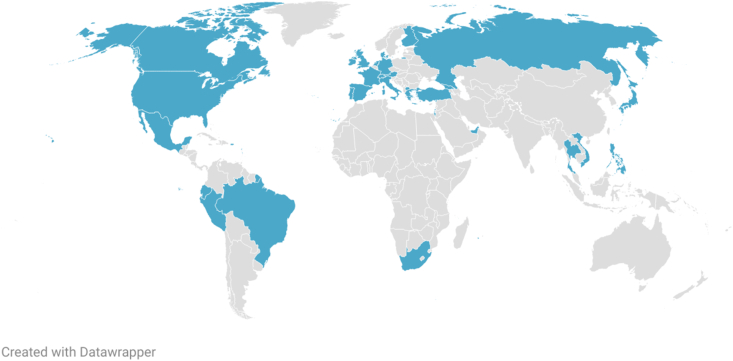
**Geographic distribution of eDelphi participants on defining response and inadequate response to TCS in AD treatment.** A world map showing the coverage of 32 countries from around the world whose experts and ADCARE members were involved in this initiative.

**Fig. 2:**
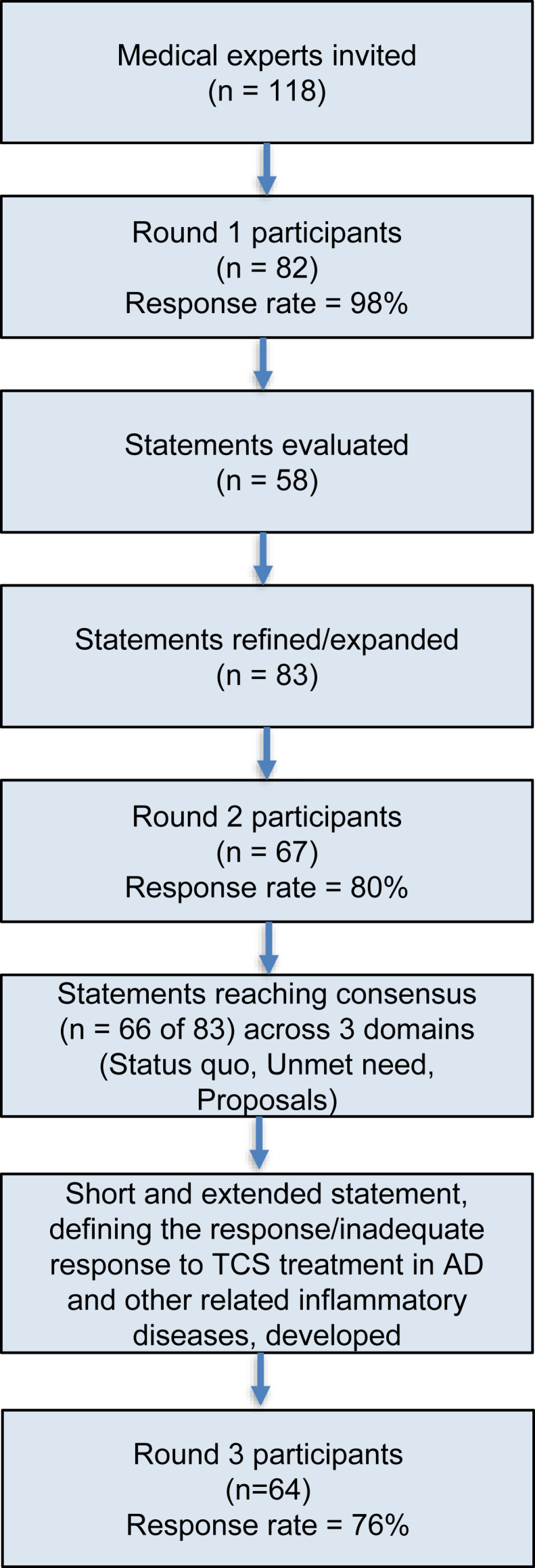
A flow diagram of the process of the eDelphi and defining statement vote. Abbreviations: EASI = Eczema Area and Severity Index; SCORAD = Scoring Atopic Dermatitis; POEM = Patient-Oriented Eczema Measure; IGA = Investigator's Global Assessment; PGA = Patient Global Assessment; NRS = Numerical Rating Scale.

A total of 83 ADCARE-certified dermatologists and allergists from 32 countries participated in the eDelphi process to develop a consensus-based definition of response and inadequate response to TCS in the treatment of AD. The geographic distribution of participating countries is illustrated in Fig. 1. The response rate was 98% in Round 1, 80% in Round 2, and 76% in Round 3. In Round 1, participants evaluated 58 statements drafted by the ADCARE steering committee and were invited to provide both quantitative ratings and written comments for clarification and refinement (results not presented). Revisions were related mostly to language framing the proposed statements, and the target threshold values remained unchanged. Based on this input, several statements were revised, merged, or expanded, and an additional set of candidate items was introduced. In total, 83 statements were circulated in Round 2. The overall flow of participants and statements across the 3 rounds is shown in Fig. 2.

Across the first 2 rounds, statements were organized into 3 predefined domains: status quo, unmet need, and proposals. Consensus, defined as ≥75% agreement (Likert scores of 4 or 5), was reached for 66 of the 83 statements. Round 2 voting served to confirm directional opinions expressed in Round 1 and to resolve areas of ambiguity or disagreement identified during the first round. No further substantial comments were submitted after Round 2, and the finalized individual consensus statements are presented below according to their respective domains.

### Status quo

Consensus was reached for 33 statements describing current practices in TCS use for AD treatment (Table 2). Experts agreed that baseline disease severity, body surface area affected, and anatomical site should guide the choice of TCS, and that potency class and licensed duration are central to safe prescribing. Agreement was also achieved on statements regarding the fundamental role of TCSs in clinical management and the principles of their use across age groups and disease severities. However, consensus was not reached on 1 statement suggesting that TCS in combination with systemic therapy should be considered first-line treatment in moderate-to-severe AD due to cost-effectiveness. Similarly, a statement suggesting that adverse effects of TCS are uncommon and reversible upon discontinuation did not meet the consensus threshold, reflecting ongoing caution among experts regarding safety perceptions.

**Table 2:**
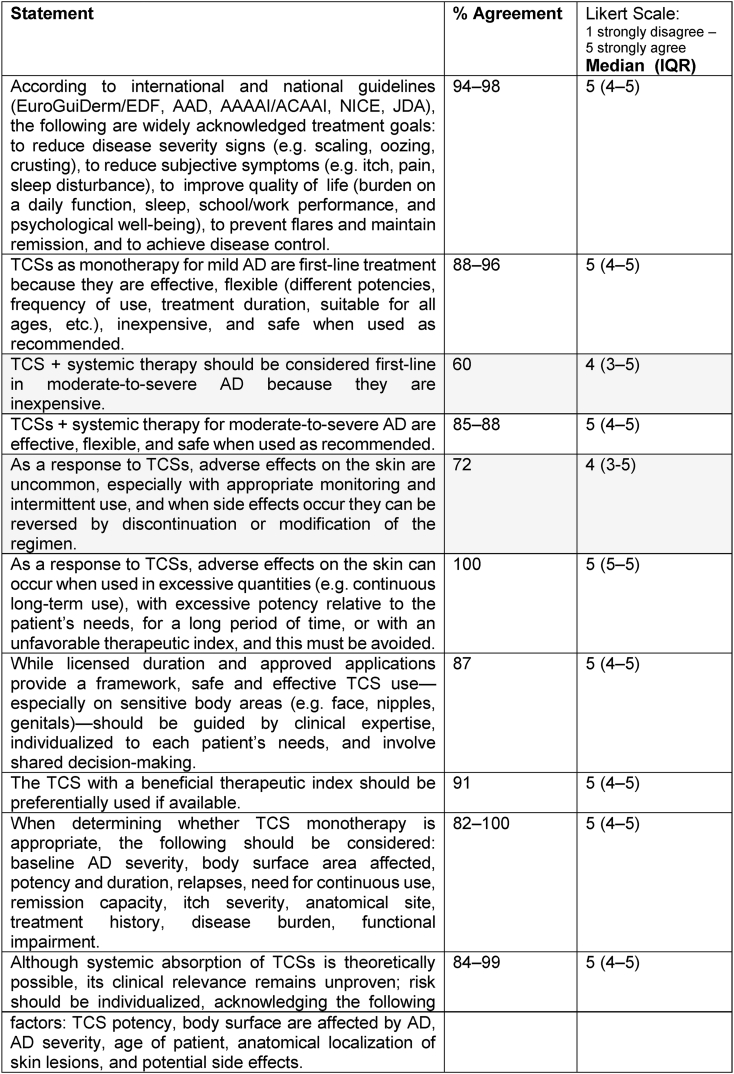
**Status quo statements on TCS use in AD (consensus in Round 2).** Consensus was defined as ≥75% agreement. Statements highlighted in grey did not reach consensus.

Abbreviations: AD = Atopic Dermatitis; TCS = Topical Corticosteroids; IQR = Interquartile Range

### Unmet need

All 12 statements addressing gaps in current practice reached consensus (Table 3). Experts identified a clear lack of standardized definitions of treatment response and inadequate response, variability in monitoring and escalation strategies, insufficient evidence for long-term management, and inconsistent integration of patient-reported outcome measures (PROMs). These findings underscore broad agreement among experts that harmonized and clinically meaningful definitions are urgently needed to improve both patient care and the comparability of clinical trials.

**Table 3:** **Unmet need statements on TCS use in AD (consensus in Round 2).** Consensus was defined as ≥75% agreement.

Statement	% Agreement	Likert Scale: 1 strongly disagree – 5 strongly agree Median (IQR)
There is a need for a standardized, objective definition of TCS treatment response/non-response	93	5 (4–5)
TCS response should be assessed based on clinical improvement within a timeframe appropriate to disease severity, anatomical site, and formulation used, rather than relying strictly on standardized durations that may not reflect individual therapies.	96	5 (4–5)
The definition of response should consider AD severity assessed by a validated disease severity score (eg, EASI, SCORAD, etc.) and TCS potency.	76–97	4–5 (4–5)
The following validated disease severity scores with predefined disease improvement thresholds are useful to define treatment response in AD with TCSs: EASI, POEM, IGA, PGA, SCORAD, NRS itch, ADCT.	78–91	5 (4–5)
The long-term safety duration of specific-potency TCSs remains uncertain across different patient populations, highlighting the need for prospective studies and individualized risk-benefit assessment in clinical care.	78	4 (4–5)

Abbreviations: AD = Atopic Dermatitis; TCS = Topical Corticosteroids; IQR = Interquartile Range; EASI = Eczema Area and Severity Index; SCORAD = Scoring Atopic Dermatitis; POEM = Patient-Oriented Eczema Measure; IGA = Investigator's Global Assessment; PGA = Patient Global Assessment; NRS = Numerical Rating Scale; ADCT = Atopic Dermatitis Control Tool

### Proposal

Twenty-one statements on proposed definitions of TCS response and inadequate response reached consensus (Table 4). Experts agreed that treatment response to TCS should be evaluated using relative improvement thresholds of at least 50% across standard outcome measures, including EASI, SCORAD, itch NRS, IGA, PGA, and POEM. Consensus also supported 14 days as a meaningful initial evaluation point for treatment response. However, absolute cut-offs (eg, EASI ≤7, SCORAD ≤25, ≥4-point POEM reduction, ≥2-point PGA reduction) did not achieve consensus, nor did very short (7 days) or long (3 months) evaluation timeframes. Similarly, there was no agreement on defining inadequate response solely based on flares (eg, ≥1 flare/month or any flare despite treatment). Statements proposing immediate escalation to systemic therapy after 7–14 days without response also failed to meet consensus, while more moderate timeframes, such as 1 month, were viewed more favorably. Overall, experts favored relative improvement metrics, clinically realistic evaluation periods, and nuanced escalation strategies rather than rigid, absolute rules.

**Table 4:**
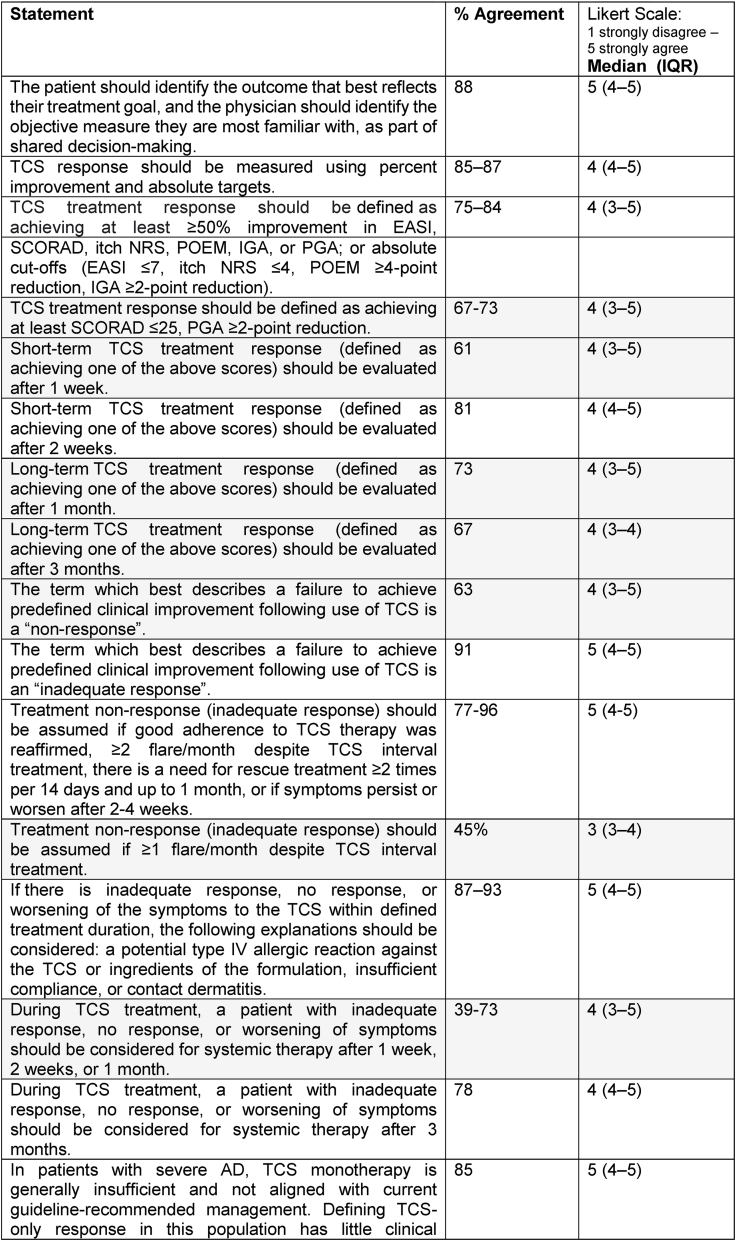
**Proposal statements on TCS response and inadequate response definitions (consensus in Round 2)**. Consensus was defined as ≥75% agreement. Statements highlighted in grey did not reach consensus.

Abbreviations: AD = Atopic Dermatitis; TCS = Topical Corticosteroids; IQR = Interquartile Range; EASI = Eczema Area and Severity Index; SCORAD = Scoring Atopic Dermatitis; POEM = Patient-Oriented Eczema Measure; IGA = Investigator's Global Assessment; PGA = Patient Global Assessment; NRS = Numerical Rating Scale; ADCT = Atopic Dermatitis Control Tool

### Consensus-based definition of TCS response and inadequate response

**Fig. 3:**
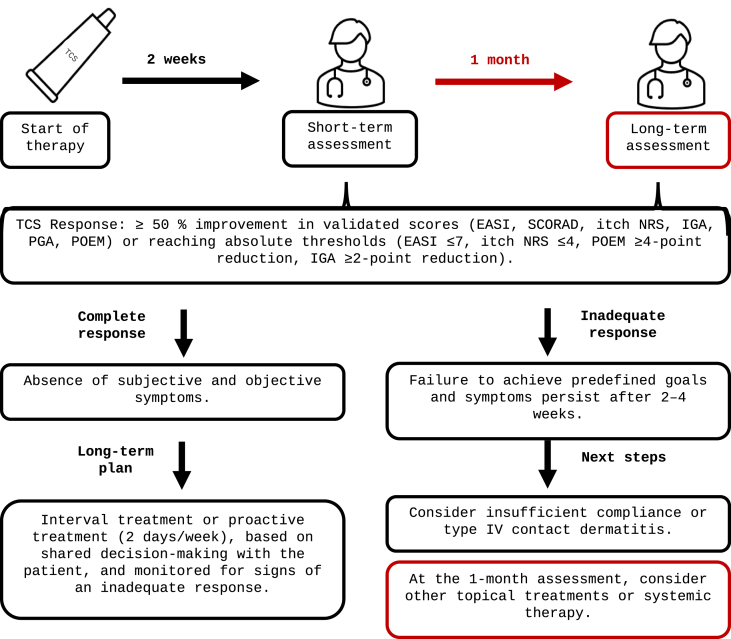
A summary of the definition of response and inadequate response to topical corticosteroid therapy in atopic dermatitis and related skin inflammatory diseases, illustrating clinical assessment time points and proposed next steps in treatment.

Following Round 2, which established clear consensus trends on key aspects of treatment response and inadequate response to TCS in AD, the final synthesis phase consolidated these findings. The resulting short and extended definitions (Table 5) were voted on in a third and final eDelphi round. These definitions are also graphically presented in Fig. 3 and stated below, capturing the agreed parameters of TCS response, applicable to AD and, in principle, to other inflammatory dermatological diseases.

**Table 5:**
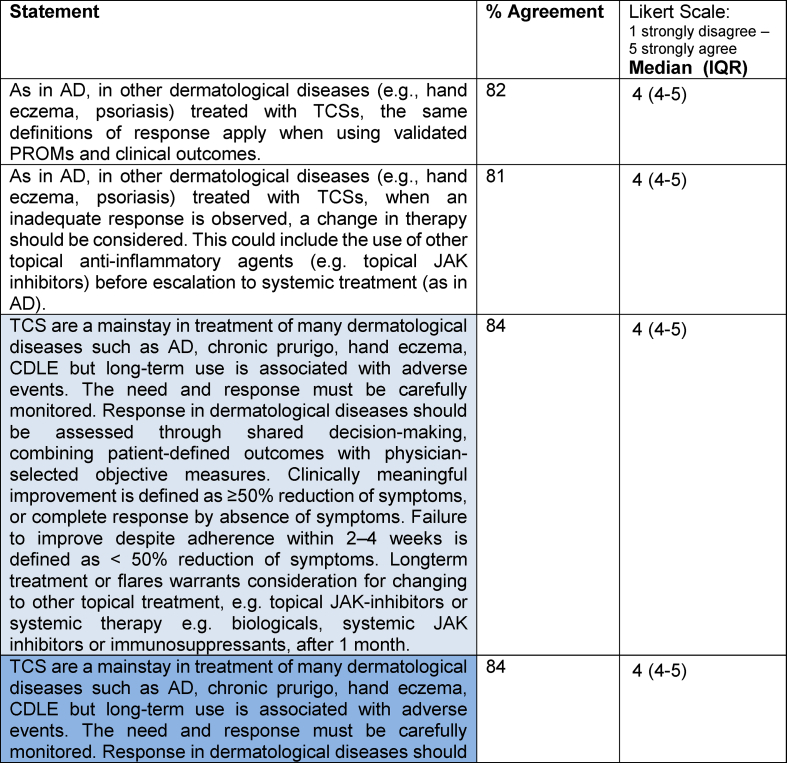

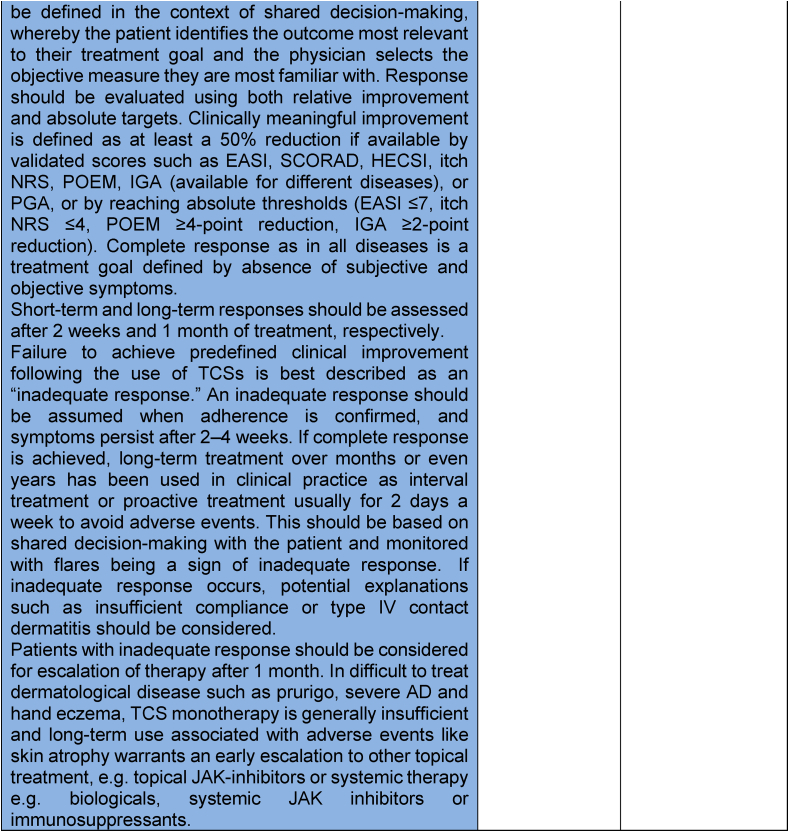
**Final statements on TCS response and inadequate response definitions in the treatment of atopic dermatitis and other related skin inflammatory diseases (consensus in Round 3)**. Consensus was defined as ≥75% agreement. The final definition statement – the short version is represented in light blue, and the extended version is represented in darker blue.

Abbreviations: AD = Atopic Dermatitis; TCS = Topical Corticosteroids; IQR = Interquartile Range; PROMs = Patient Reported Outcome Measures; CDLE = Chronic Discoid Lupus Erythematosus; JAK = Janus kinase; EASI = Eczema Area and Severity Index; SCORAD = Scoring Atopic Dermatitis; HECSI = Hand Eczema Severity Index; POEM = Patient-Oriented Eczema Measure; IGA = Investigator's Global Assessment; PGA = Patient Global Assessment; NRS = Numerical Rating Scale; ADCT = Atopic Dermatitis Control Tool

## Discussion

This initiative addresses an important gap in the standardized evaluation of a TCS response in AD and related skin inflammatory diseases. This requires consideration of both conceptual and practical aspects, linking measurable outcomes to day-to-day clinical decision-making. Some goals, such as symptom relief, can be assessed directly in routine practice, whereas others, like barrier restoration, remain more challenging to quantify and are often more relevant for long-term disease control.

Flexibility is often described as 1 of the major advantages of TCS therapy, yet the term requires precise framing. Flexibility encompasses the availability of multiple potencies, formulations, and licensed durations that permit tailoring to disease severity, anatomical site, climatic conditions, and patient age. Depending on the disease and its phase, TCS may be used for short-term induction therapy during acute flares, for intermittent or proactive maintenance to prevent relapse, or as long-term interval therapy in chronic inflammatory skin diseases such as hand eczema.^21^ Importantly, the Round 3 consensus of this eDelphi vote confirmed that such flexibility should always be accompanied by clear communication about safe and inappropriate use, particularly on sensitive areas such as the face or genitals, where clinical judgment is essential. Thus, flexibility should not be mistaken for a lack of structure but rather understood as guided adaptability within evidence-based safety limits. A practical example of this approach is reflected in the Atopic Dermatitis Integrated Care Pathways, where communication and education across all stakeholders, including specialists, general practitioners, pharmacists, and patients, are highlighted as essential to ensure consistent and safe TCS use.^22^^,^^23^

Adverse effects associated with TCS use should likewise be carefully considered and communicated. Local side effects such as iatrogenic rosacea,^24^ perioral dermatitis,^25^ persistent erythema or skin atrophy^26^ can occur and should be avoided. According to the expert opinion, when these side effects occur, they are usually mild, superficial, and reversible after modification or discontinuation of treatment. In addition, it is important to distinguish atrophy from striae, as this distinction is essential to avoid perpetuating steroid phobia.^27^ Objective tools such as dermoscopy or ultrasound can verify local changes and reinforce transparent risk communication with patients.^28^^,^^29^ While the panel agreed that appropriate TCS use is safe, ongoing anxiety among patients and clinicians highlights the need for balanced education that emphasizes both the reassuring safety profile under proper use and the potential risks of misuse or prolonged unsupervised therapy. Moreover, most restrictions on duration or site potency are based on historical caution rather than contemporary evidence. For example, in lichen sclerosus, potent agents may be used safely on genital skin for longer than 4 weeks, provided that therapy is well monitored.^30^ Product information leaflets typically recommend use “up to four weeks,” yet clinicians often extend this period briefly during acute exacerbations when benefits clearly outweigh risks. Consequently, pragmatic adjustment supported by informed patient dialogue and clinical judgment remains an essential feature of responsible care.

In evaluating efficacy, the panel reached consensus that improvement should generally be visible within about 14 days when an adequate potency is used and adherence is ensured, with re-evaluation at 1 month before considering escalation. Both seven-day and three-month assessments were regarded as impractical extremes. Furthermore, subjective improvements such as itch relief often appear before visible lesion clearance. Relative measures such as a 50% improvement in EASI, SCORAD, or similar indices provide useful reference points, but absolute cut-offs like an EASI of 7 or an itch NRS of 4, or combined physician- and patient-reported outcomes, may offer a more clinically meaningful definition of response. Future work should also focus on the cross-cultural validation of patient-reported measures to ensure international applicability.

Another aspect discussed was the concept of a therapeutic index, which is the ratio between the dose with clinical benefit and the dose at which side-effects are observed, thus reflecting the balance between efficacy and adverse effects.^31^ The panel regarded it as a helpful clinical guide rather than a prescriptive rule. Comparative data between agents of similar potency, such as mometasone and betamethasone, remain limited, and much of the current ranking of TCS potencies is based on pharmacologic extrapolation rather than controlled evidence.^32^ Therefore, the panel emphasized the need for head-to-head studies to quantify these differences and refine future guidance on risk–benefit relationships.

Despite decades of clinical experience, the literature still lacks high-quality longitudinal data stratified by potency, age group, and anatomical site. Existing thresholds for safe duration largely rely on theoretical assumptions or extrapolations from systemic corticosteroids. To address this gap, future studies should focus on prospective, population-specific safety evaluations and on pharmacovigilance registries that capture cumulative exposure, adherence, and skin integrity outcomes over time. In parallel, developing composite response indices that combine objective signs, patient-reported outcomes, flare frequency, and remission duration would provide more clinically meaningful and reproducible definitions of TCS effectiveness.

In summary, this consensus, finalized through 3 Delphi rounds, provides a framework for defining response and inadequate response to TCS therapy in AD. By combining validated clinical measures, patient-reported outcomes, and pragmatic timelines, these definitions offer a harmonized foundation for both routine care and clinical trials. Ultimately, they highlight remaining evidence gaps in long-term safety and comparative potency and underscore the need for continued collaborative research to refine and update future guidance in this essential area of therapy. These definitions may help guide future real-world research and clinical trials, and their practical applicability will be evaluated in upcoming ADCARE research initiatives.

## Abbreviations

AAD: American Academy of Dermatology; AAAAI: American Academy of Allergy, Asthma & Immunology; ACAAI: American College of Allergy, Asthma & Immunology; AD: Atopic Dermatitis; ADCARE: Atopic Dermatitis Centre of Reference and Excellence; ADCT: Atopic Dermatitis Control Tool; AEs: Adverse events; CDLE: Chronic Discoid Lupus Erythematosus; DLQI: Dermatology Life Quality Index; EASI-75: Eczema Area and Severity Index 75%; EDF: European Dermatology Forum; EuroGuiDerm: European Dermatology Forum Guideline Platform; GA2LEN: Global Allergy and Asthma Excellence Network; GISS: Global Individual Signs Score (erythema, infiltration or papulation, excoriations, and lichenification); HADS: Hospital Anxiety and Depression Scale; HECSI: Hand Eczema Severity Index; IGA: Investigator's Global Assessment; JAK: Janus kinase; JDA: Japanese Dermatological Association; NICE: National Institute for Health and Care Excellence; NRS: Numerical Rating Scale; PGA: Patient Global Assessment; POEM: Patient-Oriented Eczema Measure; PROMs: Patient-reported outcome measures; QoL: Quality of Life; SCORAD: Scoring Atopic Dermatitis; Th2: Type 2 helper T cells; TI: Therapeutic index.

## Author contributions

Conceptualization: T. Zuberbier and M.P. Pereira.

Methodology: K. Stevanovic, T. Zuberbier.

Investigation (Delphi voting): All expert authors.

Analysis of voting statements: K. Stevanovic, M.P. Pereira, T. Zuberbier.

Project administration (eDelphi coordination): K. Stevanovic, T. Zuberbier.

Visualization (tables and figures): K. Stevanovic, M.P. Pereira.

Writing – Original Draft: K. Stevanovic.

Writing – Review & Editing: All expert authors.

## Data availability

Data sharing not applicable to this article as no datasets were generated or analysed during the current study.

## Ethics statement

No human participants, identifiable data, or biological materials were involved in the development of this statement. As such, institutional ethics review and approval were not required.

## Disclosure statement regarding use of artificial intelligence

Nothing to disclose.

## Funding

This work was supported through non-institutional funding provided by the Global Allergy and Asthma Excellence Network (GA^2^LEN).

## Conflict of interest statements

**T. Zuberbier** has received institutional funding for research and/or honoria for lectures and/or consulting from Amgen, AstraZeneca, AbbVie, ALK, Almirall, Astellas, Bayer Health Care, Bencard, Berlin Chemie, Blueprint Medicines, Celltrion, FAES, HAL, Henkel, Kryolan, Leti, L'Oreal, Meda, Menarini, Merck, MSD, Novartis, Pfizer, Sanofi, Stallergenes, Takeda, Teva, UCB, Uriach, and Viatris, in addition, he is a member of ARIA, DGAKI, ECARF, GA^2^LEN and WAO. **M. Augustin** has served as a consultant, lecturer, researcher, and/or has received institutional research grants from companies manufacturing drugs for atopic dermatitis, including AbbVie, Almirall, Bayer, Beiersdorf, Eli Lilly, Galderma, Incyte, LEO Pharma, L'Oreal, MSD, Novartis, Pfizer, Regeneron, Roche-Posay, Sanofi-Genzyme, and Sun. **L. Beck** has received grants from NIAID, travel support from Sanofi, consulation fee payments from Abbvie, Amgen, Apogee, Arcutis, Arena Pharmaceuticals, Astra Zeneca, Astria Therapeutics, Bambusa Therapeutics Inc, Belharra Therapeutics, Celldex Therapeutics, Dermavent, Eli Lilly and Company, Escient Pharma, Evommune, Galderma, Gilead, GlaxoSmithKline, Invea Therapeutics, Janssen, LEO Pharma, Merck, Nektar Therapeutics, Novartis, Numab Therapeutics, Phylaxis, Pfizer, Rapt Therapeutics, Regeneron Pharmaceuticals Inc., ResVitaBio, Inc., Ribon Therapeutics, Sanofi- Aventis/Genzyme, Sitryx Therapeutics, Stealth BioTherapeutics, TRex Bio, Inc, Triveni Bio, UCB Pharma, Union Therapeutics, Xencor and Zai Laboratory, is a member of the data monitoring committee for Novartis; and owns stocks in Rapt Therapeutics. **C. Binselv-Jensen** has received consulting fee payments from Novartis, Sanofi, Celltrion, and ALK. **C. Bangert** has received honoraria from and consulting fee payments from Almirall, Novartis, Leo Pharma, Pfizer, Eli Lilly, Abbvie, Schülke Mayr GmbH, Galderma, Incyte, Sanofi. **I. Cherrez-Ojeda** received honoraria and travel fee support from Sanofi. **M.J. Cork** has received institutional research funding from Hyphens Pharma, Kenvue, Pfizer, Sanofi, L’Oréal, Leo Pharma, and Regeneron; personal fees for consulting from Hyphens Pharma, Kenvue, Procter & Gamble, Regeneron, L’Oréal, Leo Pharma, Pfizer, Sanofi, Galderma, Incyte, and UCB; honoraria for lectures from Kenvue, Regeneron, L’Oréal, Pfizer, and Sanofi and has also received support for meeting attendance from Regeneron, L’Oréal, Pfizer, and Sanofi. **T. Craig** has received research funding and/or personal fee payments as a researcher, advisor, consultant, or speaker from CSL Behring, Takeda, KalVista, Astria, Ionis, BioMarin, Pharvaris, Intellia, and BioCryst, and serves on the HAE-A medical advisory board and as an ACARE Center Director. **P.R. Criado** has received consulting fees through advisory board roles and honoraria for lectures from Novartis, AbbVie, Pfizer, and Sanofi, and has received meeting and travel support from Sanofi, Novartis, and Pfizer. **A. Custovic** has received honoraria for lectures, support for attending meetings, and is participation on advisory boards for Leo Pharma, Sanofi, Galderma, Lilly, Pfizer, Almirall, Abbvie, Incyte, and Novartis. **M.S. de Bruin-Weller** has received grants from AbbVie, Almirall, Eli Lilly, Leo Pharma, Pfizer, Regeneron and Sanofi-Genzyme, consulting fees from Almirall, Amgen, Eli Lilly, Galderma, Leo Pharma, Pfizer, Regeneron, and Sanofi-Genzyme, and honoraria from AbbVie, Almirall, Amgen, Eli Lilly, Galderma, Leo Pharma, Novartis, Pfizer, Takeda, Regeneron, and Sanofi-Genzyme**. L.F. Ensina** has received honoraria from Novartis, Sanofi, Abbvie, and travel support from Sanofi, **R. Ertaş** has received honoraria from Sanofi, Novartis, Lilly and Abbvie. **S.M. Ferrucci** has received consulting fee payments from Sanofi, Abbvie, Leo Pharma, Eli Lilly, Galderma, Almirall, honoraria from Abbvie, Almirall, Eli Lilly, Leo Pharma, Galderma, Incyte, Sanofi, Novartis, travel fee support from Almirall, Sanofi, Abbvie, Leo Pharma, and is on an advisory board for Galderma, Incyte, and Sanofi. **N. R. Fihlo** has received honoraria from Sanofi, Astra Zeneca, Chiesi, Novartis, Abbvie and travel fees support from Sanofi and Astra Zeneca. J. H. Fluhr has received payment for expert testemony from Almiral. **S. Gregoriou** has received payments for consulting, honoraria, and travel fee support from Pfizer, Abbvie, Sanofi, Lilly, Leo Pharma, Pierre Fabre, Loreal. **M. Gotua** has received support for travel fees from Berlin-Chemie and is the president of the Georgian Academy of Allergy, Asthma, and Clinical Immunology. **A. Giménez -Arnau** is or recently was a speaker and/or advisor for and/or has received research funding from Almirall, Amgen, AstraZeneca, Avene, Blue -Print, Celldex, Escient Pharmaceutials, Genentech, GSK, Harmonic Bio,Incyte, Instituto Carlos III- FEDER, Jaspers, Leo Pharma, Menarini, Mitsubishi Tanabe Pharma, Noucor, Novartis, Sanofi**–**Regeneron, Septerna, Servier, Thermo Fisher Scientific, Uriach Pharma, and is a medical Advisor for, Almirall, Amgen, Blue -Print, Celldex, Escient, FAES, Genentech, GSK, Jaspers, Leo Pharma, Mitsubishi Tanabe, Novartis, Noucor, Sanofi–Regeneron, Thermo Fisher Scientific, Septerna, Servier, Uriach Pharma Research Grants supported by ESCIENT, NOUCOR, Novartis, Instituto Carlos III- FEDER, Uriach Pharma, and participated in educational activities for Almirall, Avene, Genentech, GSK, Leo Pharma, Menarini, MSD, NOUCOR, Novartis, Sanofi, Uriach Pharma. **R. Fachini Jardim Criado** has received honoraria from Lilly, Sanofi, Pfizer, Novartis, and supporting to travel fees from Sanofi. **C. Jack** received grant payments from SkIN Canada/CIHR, Montreal Dermatology Research Institute, Canadian Dermatology Foundation, LEO, FRQS – FONDS DE RECHERCHE DU QUEBEC SANTE, MGH Foundation, BMS, Incyte, Pfizer**,** honoraria from Sanofi, LaRoche-Posay, Lilly, Johnson & Johnson, AbbVie, CDA, Novartis, McGill CPD/CPD MedUpdates, Valeant, Catalytic, Bausch, Arcutis, Pfizer, Beiersdorf, Amgen, Boehringer-Ingelheim, Incyte, Catalytic, LEO, Innomar, Galderma, Apogee Therapeutics, Chronicle Companies, LEAD, SkIN Canada, CeraVe, travel fee support from Pfizer, AbbVie, and is the scientific advisor for Eczema Quebec. **N. Katoh** received grants as an investigator from AbbVie, Boehringer Ingelheim Japan, Eisai, Eli Lilly Japan, Jansen Pharma, Kyowa Kirin, LEO Pharma, Maruho, Sun Pharma, Taiho Pharmaceutical, and Torii Pharmaceutica, and honoraria from Sanofi, Maruho, AbbVie, Eli Lilly Japan, Mitsubishi Tanabe Pharma, Jansen Pharma, Taiho Pharmaceutical, Torii Pharmaceutical, Kyowa Kirin, Celgene Japan, Otsuka Pharmaceutical, and LEO Pharma. **A. Katoulis** has received honoraria from UCB, Janssen, Pharmaserve Lilly, Amgen, AbbVie, Genesis Pharma, Leo Pharma, Pfizer, L′ Oreal, travel fee support from Janssen, Novartis, Genesis Pharma, L'Oreal, and has participated on advisory board for AbbVie, Galderma, Janssen, UCB. **D. Larenas-Linnemann** has received grant payments from Lilly, Pfizer, Abbvie, Sanofi, Bioderma/Naos, consulting payments from Lilly, honoraria from Lilly, Pfizer, Abbvie, Sanofi, Novartis, travel fee support from Pfizer and Lilly, and is chair of Mexican Atopic Derm Guidelines. **H.Y. Lee** has received royalties for chapters relating to Eczema Herpeticum, Stevens-Johnson syndrome, DRESS, speaker payments from ADCARE allergy school in Kuala Lumpur and DERM Connect in Kuala Lumpur, was an Expert witness for coroner's court, Singapore & Academy of Medicine, Singapore, received travel fee support from Sanofi, and is co-chair for the Atopic Dermatitis guidelines Singapore, a member for the BAD guidelines group for Stevens-Johnson syndrome, is part of the EADV guideline group for Stevens Johnson group, is on the EADV taskforce for Darier's disease, and in the WHO-CIOMS workgroup for severe cutaneous adverse reactions. **H. Lima** has received grant payments, honoraria, and is on an advisory board for AbbVie (Abbott), Eli Lilly, Amgen, Janssen, Aslam, La Roche-Posay, AstraZeneca, Leo Pharmaceutics, Bausch, Merck Sharp & Dohme, Bristol-Myers-Squibb, Moonlake, Celgene, Novartis, Dermira, Pediapharma, Pfizer, Sanofi, Regeneron, and received travel fee support from AbbVie (abbott), Celgene, Novartis, Pediapharma, Regeneron, and Sanofi. **E. Mansour** has received consulting fee payments from Takeda, CSL Behring, Pint Pharma, honoraria from Takeda, CSL Behring, Novartis, GSK, Pint Pharma and Sanofi, and travel fee support from Takeda, CSL Behring, Pint Pharma. V. Morris has received honoraria from Novartis, Sanofi, Abbvie, and support for travel fees from Sanofi. **A. Moschione Castro** has received honoraria from Sanofi, Abbvie, Ache, and is on an advisory board for Abbvie and Danone Nutricia. **D. Mota Veloso Bruscky** received free medication samples for patient use from GSK, Mantecorp, Farmoquimica S.A. **A. Nosbaum** has received grant payments from Sanofi, Lill, Pfizer, consulting fee payments and honoraria from Sanofi, Lilly, Medac, Leo Pharma, Pfizer, and Almirall, and is on an advisory board for Sanofi, Abbvie, Medac, Galderma and Almirall. **E. Özkaya** received grant payment from Amgen, Novartis, Sanofi Genzyme, consulting fee payments from Sanofi-Genzyme and Pfizer, and is a member of editorial advisory board of contact dermatitis (Wiley), handling editor of contact dermatitis (Wiley), member of ESCD working parties, and a member of the EADV contact dermatitis task force. **C.A.S. Parisi** has received consulting fees payments from Abbvie, Novartis, honoraria from UCB Pharma, Sanofi, travel fee support from Sanofi, Novartis and UCB Pharma. **J. Peter** has received grant payments from Takeda, Viatris, and Cipla, honoraria from Sanofi and Abbvie, and is an advisory board member for Abbvie. **L. Puig** has received Abbvie, Almirall, Amgen, Leo Pharma, Lilly, Sun Pharma and UCB, consulting fee payments Abbvie, Almirall, Amgen, Biogen, Boehringer Ingelheim, Bristol-Myers-Squibb, Fresenius-Kabi, J&J, Leo Pharma, Lilly, Novartis, Pfizer, UCB, travel fee support AbbVie, UCB, participated on an advisory board of Horizon, member of international psoriasis council, and GRAPPA. **C. Rincón-Pérez** has received consulting fee payments from Abbvie, and Lilly, honoraria from Abbvie, Lilly, Pfizer, and travel fee support from Abbvie. **U.M. Sahiner** is the president of the Turkish National Society of Allergy and Clinical Immunology. **P. Schmid-Grendelmeier** has received grant payments from Christine Kühne Center for Allergy and Education CK-CARE, consulting fee payments from AbbVie, Leo Pharma, Macro array Diagnostics, Pfizer, Unifarco, Sanofi-Regeneron, Stallergenes, ThermoFisher, honoraria from AbbVie, Leo Pharma, Macro array Diagnostics, L'Oreal, Novartis, Permamed, Pfizer, Pierre Fabre, Sanofi-Regeneron, Stallergenes, ThermoFisher, payment for expert testamony from Permamed, is on the advisory board for AbbVie, Almirall, Galderma, Leo Pharma, Sanofi-Regeneron, is the Treasurer Int Society for Atopic Dermatitis ISAD, on the scientific committee of the Swiss Center for Allergy AHA (www.aha.ch), has stocks for Derma2Go, and has received materials from Bühlmann and Euroimmun. **E. Serra-Baldrich** received honoraria for lectures, support for attending meetings and participation on Advisory Boards for Leo Pharma, Sanofi, Lilly, Pfizer, Abbvie, Incyte, Novartis. **C.H. Smith** has received research funding from academic-industry partner consortia (see HIPPOCRATES-IMI.eu, BIOMAP-IMI.eu), OPENTARGETS, Astra-Zeneca, UCB and Pfizer. **P. Staubach** has received grant payments from Sanofi, AbbVie, LEO Pharma, Novartis, UCB Pharma, Janssen Pharmaceuticals, Almirall, Kalvista, L’Oréal, Pierre Fabre, Amgen, honoraria from Sanofi, AbbVie, LEO Pharma, Pfizer, Eli Lilly, Novartis, UCB Pharma, Almirall, Pierre Fabre, Beiersdorf, Incyte, Janssen Pharmaceuticals, L’Oréal, is on advisory boards for Sanofi, AbbVie, Incyte, Pfizer, Eli Lilly, Novartis, UCB Pharma, Almirall, L’Oréal, Janssen Pharmaceuticals and was the past president of Society of Dermopharmacy. **D. Thaci** has received consulting fee and honoraria payments from AbbVie, Amgen, Almirall, Bristol-Myers-Squibb, Boehringer Ingelheim, Celltrion, Galderma, Janssen, Leo-Pharma, Lilly, L′ Oreal, New Bridge, Novartis, Pfizer, Regeneron, Roche-Possay, Sanofi, Takeda, UCB und Vichy, is on the scientific and registry steering committee of the German Psoriasis Association. M.J. Torres has received grant payments from ISCIII, SEAIC, European Commission, and thr Andalusian Government, consulting fee payments form Diater, Leti Laboratories, Aimmune Therapeutics, honoraria from Diater, Leti Laboratories, and Aimmune Therapeutics, and travel fee support from EAACI. **T. Torres** has received consulting pee payments from AbbVie, Almirall, Apogee, Boehringer Ingelheim, Bristol Myers Squibb, J&J, LEO Pharma, Eli Lilly, Novartis, Pfizer, Sanofi-Genzyme, Sandoz, STADA, UCB, honoraria from AbbVie, Almirall, Boehringer Ingelheim, Bristol Myers Squibb, J&J, LEO Pharma, Eli Lilly, Novartis, Pfizer, Sanofi-Genzyme, Sandoz, STADA, UCB, and is on an advisory board for Sanofi. **P. Tuchinda** has received honoraria from Novartis, A. Menarini, and Sanofi. **G.V Duarte** has received honoraria from Abbvie, Jansen, Amgen, Celldex, Sanofi, Lilly, Pfizer, Galderma, Sun Pharma, and BMS, travel fee support from UBC, Sanofi, Janssen, Lilly, is on advisory boards for Pfizer, Galderma, and Sun Pharma. **H. Virtanen** has received consulting fee payments from Abbvie and Novartis, honoraria from UCB Pharma, and Sanofi, and has received travel fee support from Sanofi, Novartis, and UCB. **T. Werfel** reports institutional grants or personal fees for lectures or advisory boards from AbbVie, Almirall, Beiersdorf, Eli Lilly, Galderma, Janssen/JNJ, Leo Pharma, Novartis, Pfizer, Sanofi-Regeneron. **A. Wollenberg** received honoraria from Abbvie, Almirall, AMGEN, Beiersdorf, Bioderma, DKSH, Galderma, Glenmark, Jamjoom, Leo Pharma, Eli Lilly, L'Oreal, Maruho, Novartis, Pfizer, Pierre Fabre, Regeneron, and Sanofi-Aventis, received travel fee support from Abbvie, Leo Pharma, Pierre Fabre, is on advisory boards for Abbvie, Aileens, Almirall, Amgen, Bioproject, BMS, Galapagos, Galderma, GSK, Hans Karrer, Hexal, Janssen, Kyowa Kirin, Leo Pharma, Eli Lilly, L'Oreal, Maruho, Merck, Novartis, Pfizer, Pierre Fabre, Regeneron, Sandoz, Sanofi-Aventis, Schülke&Mayr and UCB, is the executive board member of the International Society of Atopic Dermatitis, and is the Vice Chair of the European Task Force on Atopic Dermatitis, and has received clinical trial support from Abbvie, Almirall, Galderma, Leo Pharma, Eli Lilly, L'Oreal, Merck, Novartis, Pfizer, Pierre Fabre, Regeneron, Sanofi-Aventis and UCB. **M. Worm** has received consulting fee payments and honoraria from AbbVie, Aimmune Therapeutics, ALK-Abelló, Allergopharma, Almirall, Amge, AstraZenceca, Bayer, Bencar, Bioprojet Pharma, Boehringer Ingelheim, Bristol Myers Squib, Galderm, Glaxo Smith Klin, Infectophar, LEO Pharma, Lilly, Mylan Germany, Novartis, Octapharm, Pfize, Sanofi-Aventis Genzyme and Stallergenes. Y. Weng Yew received travel fee support from Elli Lilly. **M. P. Pereira** received research funding from Almiral, Clinuvel, Pfizer, cosnulting fee payments from Almirall, Galderma, Incyte, Sanofi, honoraria from AbbVie, Beiersdorf, Doctorfix, Eli Lilly, Falk, Foundation, FomF GmbH, GA2LEN, Galderma, Novartis, P.G. Unna Academy, Sanofi, StreamedUp, TouchMDT, Yai Lab, has received travel fee support from AbbVie, Celltrion, CSL Behring, GA2LEN, Galderma, Sanofi, and was an investigator for Allakos, Celldex Therapeutics, Escient, Incyte and Sanofi. **M. Abuzakouk, I. Angelova Fischer, L.K. Arruda, J. Bernstein, M. Bertolín-Colilla, O. Calderón-Llosa, G. W.Canonica, M.A.R. Castor, R.J. Casis Hao, K.J.L. Choo, L. Chularojanamontri, T. Çetinarslan, F. Çalıcıoğlu, N. Douladiris, Y. Erdem, C. Flohr, D. Fomina, W. Hoetzenecker, C.G. Mortz, M. Morelo Rocha Felix, O. Mukhina, D. Van Nguyen, D. Ozceker, R Oztas Kara, G.D. Ramon, B. Sevimli Dikicier, K. Stevanovic, O. Su Kucuk, N. Teovska Mitrevska, M.E. Trócoli Zanetti, Z. Vadasz, E. Vakirlis** declare no conflict of interest.
